# Risk of Renal Function Decline in Patients with Ketamine-Associated Uropathy

**DOI:** 10.3390/ijerph17197260

**Published:** 2020-10-04

**Authors:** Shih-Hsiang Ou, Ling-Ying Wu, Hsin-Yu Chen, Chien-Wei Huang, Chih-Yang Hsu, Chien-Liang Chen, Kang-Ju Chou, Hua-Chang Fang, Po-Tsang Lee

**Affiliations:** 1 Division of Nephrology, Department of Internal Medicine, Kaohsiung Veterans General Hospital, Kaohsiung 813, Taiwan; blueyeou1104@gmail.com (S.-H.O.); xychen16@gmail.com (H.-Y.C.); cwhuang0824@vghks.gov.tw (C.-W.H.); cyhsu@vghks.gov.tw (C.-Y.H.); cclchen@seed.net.tw (C.-L.C.); kjchou@vghks.gov.tw (K.-J.C.); hcfang@vghks.gov.tw (H.-C.F.); 2 Faculty of Medicine, School of Medicine, National Yang-Ming University, Taipei 112, Taiwan; 3 Graduate Institute of Clinical Medicine, College of Medicine, Kaohsiung Medical University, Kaohsiung 807, Taiwan; 4 Department of Obstetrics and Gynecology, Kaohsiung Chang Gung Memorial Hospital, Kaohsiung 833, Taiwan; wudoudou123@cgmh.org.tw

**Keywords:** hydronephrosis, ketamine-associated uropathy, renal function decline

## Abstract

Ketamine-associated diseases have been increasing with the rise in ketamine abuse. Ketamine-associated uropathy is one of the most common complications. We investigated the effects of ketamine-associated uropathy on renal health and determined predictors of renal function decline in chronic ketamine abusers. This retrospective cohort study analyzed 51 patients (22 with ketamine-associated hydronephrosis and 29 with ketamine cystitis) from Kaohsiung Veterans General Hospital in Taiwan. Primary renal outcome was end-stage renal disease or estimated glomerular filtration rate decline >30% from baseline. Compared with the ketamine cystitis group, the hydronephrosis group had lower initial and final estimated glomerular filtration rates and higher alkaline phosphatase and gamma-glutamyl transferase levels (*p* < 0.05). Elevated cholestatic liver enzyme levels correlated with renal dysfunction in ketamine-associated uropathy. The hydronephrosis group had a higher proportion of patients reaching endpoints than the ketamine cystitis group (50% and 7%, respectively, *p* < 0.001). After adjusting for age, sex, and initial serum creatinine level, hydronephrosis remained an independent risk factor for renal function deterioration. Ketamine-associated hydronephrosis was a poor renal outcome and strong predictor of renal function decline in chronic ketamine abusers. Elevated cholestatic liver enzyme levels correlated with the severity of ketamine-associated uropathy. Ultrasonography screening of these high-risk groups and regular renal function follow-ups are necessary.

## 1. Introduction

Ketamine, a non-competitive N-methyl-D-aspartate (NMDA) receptor antagonist, was first synthesized in the United States in 1962. Since then, it has been widely used as an anesthetic and analgesic agent in human and animal medicine. In addition, it has been adopted as an agent in the treatment of major depression, treatment-resistant depression, and bipolar affective disorder [1,2]. However, given its psychostimulant properties and hallucinogenic effects, ketamine has been a globally popular, illegal recreational drug among young people since the 1980s [3]. In the past decade, ketamine abuse has become a problem of serious social concern with increasing prevalence due to its highly addictive nature. In addition, the reported events of ketamine abuse in Taiwan have increased from 200 to 2904 cases between 2008 and 2016. Currently, ketamine is officially classified as a Schedule III controlled substance in both the United States and Taiwan [4,5].

Ketamine has been previously used as a dissociative anesthetic agent because of its central nervous system effect and complex neuropharmacological action. The hypnotic effect of ketamine appears to be mainly mediated by N-methyl-D-aspartate (NMDA) and hyperpolarization-activated cyclic nucleotide-gated channel 1 (HCN1) receptor blockades. Additionally, ketamine induces psychotomimetic, analgesic, and antidepressant effects through delayed and prolonged neuromodulation and cell signaling cascades [6]. Ketamine users may appear dissociated from environmental reality, often experiencing auditory hallucinations [7]. Ketamine is a highly lipid-soluble material that undergoes rapid catabolism and redistribution into the peripheral tissues following its intravenous injection into the human body [8]. It is metabolized in the liver by N-demethylation and ring hydroxylation pathways into active metabolites, mainly “norketamine”, which is one-third to one-fifth as potent as ketamine itself. Most of the ketamine metabolites are excreted into urine by the kidney; only 1% to 5% is eliminated via fecal excretion. Usually, the elimination half-life of ketamine ranges between 2 and 3 hours in adults; however, it is occasionally detected in the urine up to 2 weeks after use [9].

Ketamine abuse causes ketamine-associated complications, such as genitourological, neuropsychiatric, hepatobiliary, and gastrointestinal complications [10]. Initial case series reports of ketamine-associated cystitis were published in 2007 [11,12]; lower urinary tract symptoms, including frequency, urgency, dysuria, nocturia, urge incontinence, and even painful gross hematuria, were common complaints of chronic ketamine abusers.

After efforts and research by urologists, pathologists, pharmacologists, and toxicologists, there is now a more distinct pathophysiology of ketamine-associated cystitis. However, the toxic effect of ketamine on the upper urinary tract and kidney is not clearly understood. Chu et al. demonstrated that ketamine-associated uropathy involved both the lower and upper urinary tracts, and 51% of the analyzed patients had unilateral or bilateral hydronephrosis, as revealed by ultrasonography [11]. If the upper urinary tract is involved, percutaneous nephrostomy might be necessary to relieve obstruction and preserve renal function [13]. However, the exact side effects of ketamine-associated hydronephrosis are difficult to assess. To date, few studies have focused on the long-term impacts of ketamine-associated uropathy on renal function; thus, this retrospective study aimed to investigate the influence of ketamine-associated uropathy on renal function and determine the risk factors for renal failure in chronic ketamine abusers.

## 2. Materials and Methods

### 2.1. Study Design and Patient Population

A retrospective cohort study was conducted between January 2008 and October 2019 in Kaohsiung Veterans General Hospital, Taiwan. Data of chronic ketamine abusers presenting with ketamine-associated uropathy were collected through a chart review to investigate the risk of renal function deterioration. The ethics committee of Kaohsiung Veterans General Hospital approved (IRB No: KSVGH20-CT2-10) and supervised the study in accordance with the tenets of the Declaration of Helsinki (1975) and its later amendment (2013). The requirement of informed consent was waived by the aforementioned ethics committee due to the retrospective nature of this study.

Patients aged ≥20 years who were current or former ketamine abusers with lower urinary tract symptoms at the time of assessment were included. Exclusion criteria were an age <20 years; not having undergone ultrasonography or abdominal computed tomography (CT) at our hospital; not having undergone regular renal function follow-up examinations after discharge; exhibiting other disease-related abnormalities in kidney anatomy; and confirmed known glomerulopathy, interstitial nephritis, or tubulonephritis before admission. Ketamine-associated uropathy was diagnosed based on self-reported drug use history, symptoms, imaging study, and clinical exclusion of other possible etiologies.

### 2.2. Baseline Characteristics and Radiology Images

Data on baseline demographics and underlying comorbidities were collected for each included patient. Chronic comorbidities, such as hypertension and type 2 diabetes mellitus, were defined using medical records of patients on regular treatment medication for >3 months. In addition, laboratory data and radiologic findings were recorded. Hydronephrosis was defined as dilatation of the collecting system in one or both kidneys, whereas cystitis was defined as clinically exhibiting lower urinary tract symptoms without hydronephrosis. All radiological images were analyzed by a competent radiologist via ultrasonography or abdominal CT.

### 2.3. Clinical Outcomes

The primary outcome was the occurrence of end-stage renal disease (ESRD), or an estimated glomerular filtration rate (eGFR) decline >30% of baseline. eGFR was calculated using a modified equation of the Modification of Diet in Renal Disease Study. ESRD was defined as an irreversible decline in renal function and a lifelong dependence on dialysis. Another renal outcome surrogate was an eGFR decline >30% of baseline, which was proved by many studies to be strongly associated with the risk of subsequent ESRD occurrence or death [14,15]. All patients were followed up with until death, until 31 October 2019, or until they were lost during the follow-up examination period.

### 2.4. Statistical Analyses

Data are appropriately expressed as means ± standard deviation or number (percentage). Groups were compared using the independent Student’s *t*-test and χ^2^-test for normally distributed continuous and categorical variables, respectively. Non-parametric data were compared using the Mann–Whitney U test. The association between the difference in ketamine-associated uropathy and renal outcomes was estimated using the Kaplan–Meier curve assessment. In addition, significant determinants of renal function deterioration were evaluated using Cox regression models to estimate the effects of hydronephrosis after controlling for age, sex, and initial renal function. Statistical significance was set at a two-tailed *p* < 0.05, and all statistical analyses were performed using the SPSS statistical software 22.0.0 (IBM Corp., Armonk, NY, USA).

## 3. Results

**Figure 1:**
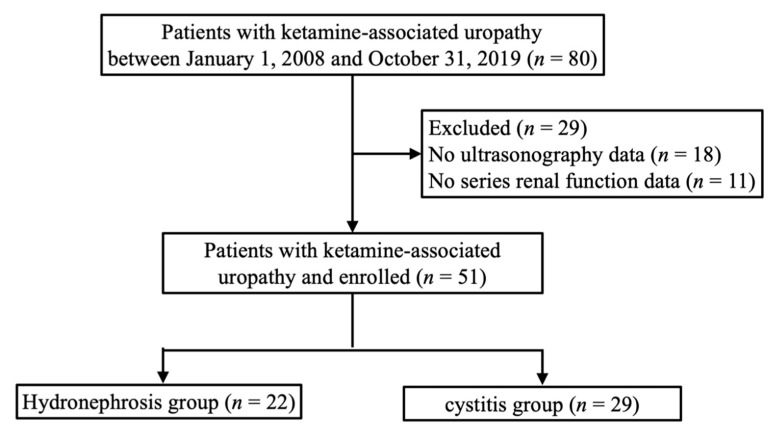
Flow chart of participants in the cohort study.

Of the 80 patients who presented with ketamine-associated uropathy, as shown in Figure 1, 18 did not have available ultrasonography results and 11 did not undergo renal function follow-up examinations after discharge; therefore, these patients were excluded. The included patients were categorized into two groups as follows: patients with ketamine-associated hydronephrosis and patients with only ketamine cystitis. Patients in the former group were those with hydronephrosis on presentation, not relating to other etiologies of structural abnormality. By contrast, patients in the latter group were those who exhibited only lower tract urinary symptoms without hydronephrosis. In total, 51 patients classified into the ketamine-associated hydronephrosis (*n* = 22) and ketamine cystitis (*n* = 29) groups were included in this study. Table 1 shows their clinical characteristics and biochemical parameters. There were 14 male and 37 female patients (mean age, 35.29 ± 5.08 years), with a slight predominance of female patients. The patients were relatively thin and malnourished compared with the general population, with an average body mass index (BMI) of 19.92 ± 3.91 kg/m^2^. Three patients had an underlying comorbidity with hypertension, and two patients had type 2 diabetes mellitus.

Additionally, Table 1 illustrates the comparison between the hydronephrosis group and ketamine cystitis group. No significant differences regarding sex, age, body height, body weight, BMI, systolic blood pressure, and diastolic blood pressure were observed between the two groups. The initial and final eGFRs were significantly lower in the hydronephrosis group than in the ketamine cystitis group (*p* < 0.05). Regarding liver function, an inconsiderable difference was noted in glutamic oxaloacetic transaminase, glutamic pyruvic transaminase, and total bilirubin levels in these groups; however, significantly higher alkaline phosphatase and gamma-glutamyl transferase levels were observed in the hydronephrosis group than in the cystitis group (*p* < 0.05). The elevated cholestatic liver enzyme levels correlated with the degree of renal dysfunction in terms of the initial, worse, and final eGFRs throughout the course of ketamine-associated uropathy in these patients, as shown in Table 2.

**Figure 2:**
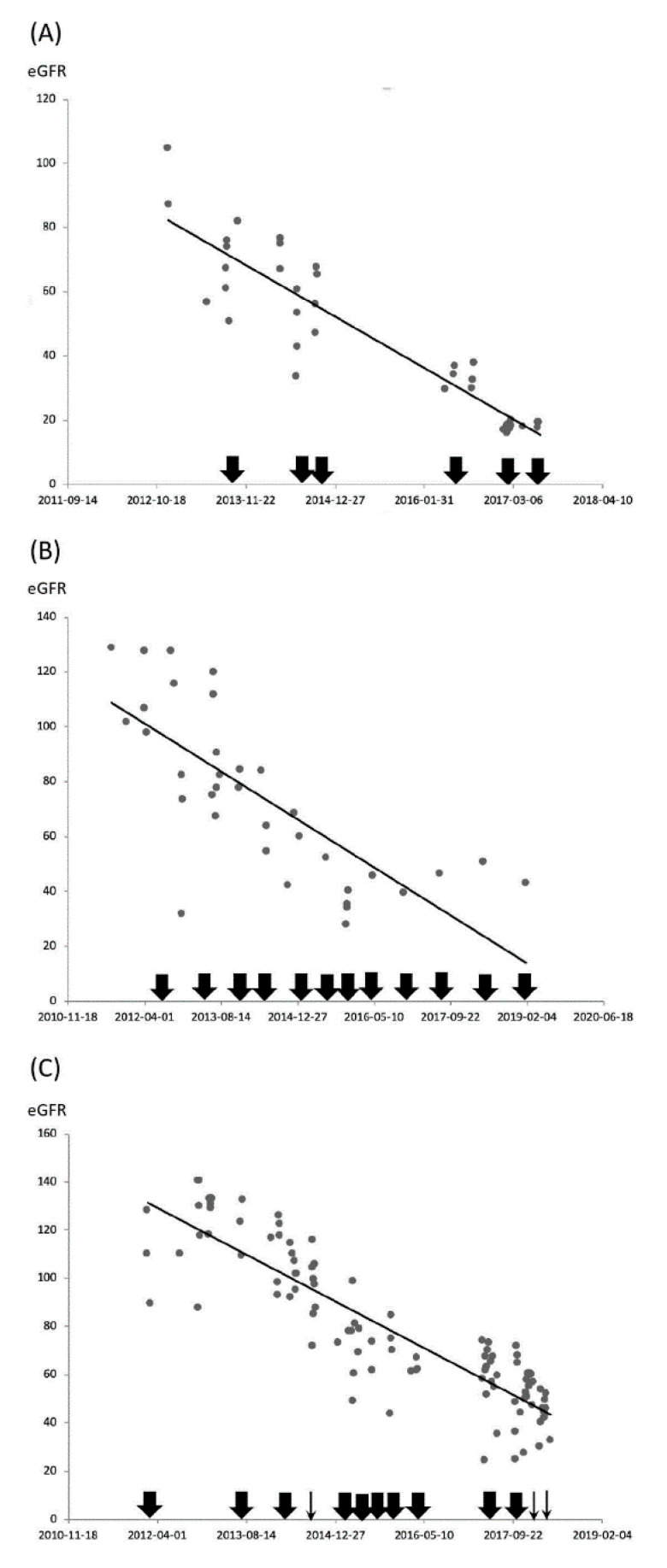
Changes in renal function in three patients with ketamine-associated hydronephrosis. There is an unavoidable deterioration of renal function following repeated recovery from urinary tract obstruction and control of infection. (**A**) A 29-year-old woman had been repeatedly hospitalized due to recurrent episodes of urinary tract infection and hydronephrosis. She was effectively treated with appropriate antibiotics and insertion of double-J stents during each episode. (**B**) A 27-year-old woman underwent regular double-J stent exchanges every 6 months. (**C**) A 24-year-old man was frequently admitted due to recurrent episodes of hydronephrosis and acute kidney injury, and was subjected to double-J stent exchange or percutaneous nephrostomy to reduce the obstruction each time. A thick arrow represents double-J stent intervention, a thin arrow depicts percutaneous nephrostomy. eGFR, estimated glomerular filtration rate.

**Figure 3:**
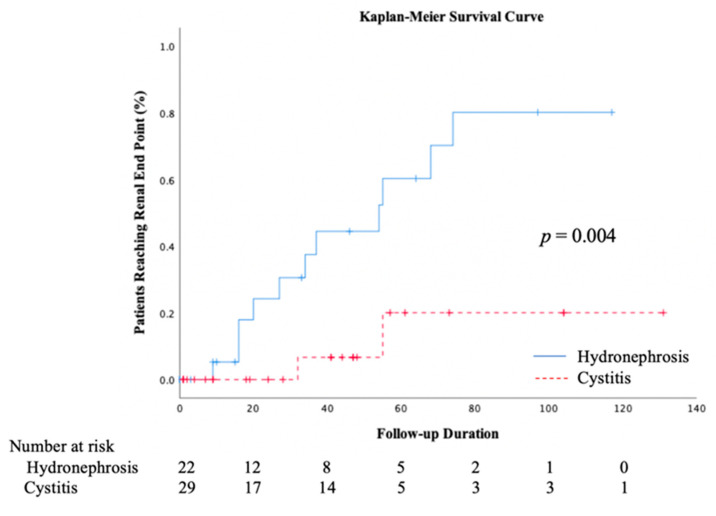
Progression of ketamine uropathy by hydronephrosis status. The Kaplan–Meier curves show the cumulative probability of reaching the renal endpoint of a 30% decline in the glomerular filtration rate or progression to end-stage renal disease among patients with ketamine uropathy, stratified according to hydronephrosis status.

In addition, renal function was monitored via longitudinal follow-up examinations. Patients with hydronephrosis were divided into three groups according to their change in renal function. According to our results, 15 patients displayed gradually deteriorated renal function, 2 displayed stable renal function, and 5 displayed improved renal function. Figure 2 features the changes in renal function of the patients with ketamine-associated hydronephrosis and gradually deteriorated renal function. Although every effort had been made to treat episodes of infection and relieve urinary obstructions through optimal intervention, the decline in renal function remained inevitable. During the median follow-up of 37 months, 11 patients in the hydronephrosis group and 2 in the cystitis group reached the primary renal outcome (50% and 7%, *p* < 0.001). Two of the 11 patients who matched the renal outcome in the hydronephrosis group progressed to an ESRD status and were subjected to hemodialysis. Conversely, no patient in the cystitis group was subjected to dialysis during the follow-up period. The Kaplan–Meier curve in Figure 3 illustrates the significant difference in renal survival between the two groups (Log-rank test, *p* = 0.004).

Table 3 presents the results of the multivariate analysis to evaluate possible risk factors associated with renal outcome among ketamine-associated uropathy patients. The occurrence of hydronephrosis remained an independent risk factor for renal function deterioration, even after adjusting for age, sex, and initial creatinine level. Among ketamine-associated uropathy patients, the risk of renal function deterioration was 5 times higher for patients with hydronephrosis than for those with cystitis alone (95% confidence interval, 1.128 to 25.137).

## 4. Discussion

This study evaluated the effects of ketamine-associated uropathy on renal health and the clinical difference between patients with upper urinary tract symptoms and those with lower urinary tract symptoms. This study had three major findings. First, chronic ketamine abusers with ketamine-associated hydronephrosis had a worse renal outcome than those with cystitis alone. This association remained significant even after adjusting for confounding factors. Second, hydronephrosis in patients with ketamine-associated uropathy might indicate progression to an irreversible stage and inevitable deterioration in renal function. Third, the extent of hepatobiliary damage, mainly with the elevation of cholestatic liver enzyme levels, correlated with the severity of renal dysfunction in chronic ketamine abusers.

Since ketamine is mainly excreted by urine, the side effect of ketamine on the urological system is commonly reported [16,17]. The exact mechanism of ketamine-associated cystitis is complicated. Most studies have suggested that major injury results from the direct toxic effects of ketamine and its metabolites on bladder tissue, including denuded bladder mucosa and decreased expression of tight junction protein in the bladder barrier [18,19]. Other hypotheses such as microvascular ischemia of the bladder [11], neurotransmitter dysregulation inducing detrusor overactivity [20], and IgE-mediated hypersensitivity [21] may also play important roles in the occurrence of ketamine-associated cystitis. These factors caused chronic inflammation and induced fibrosis reaction, eventually resulting in an ulcerative and contracture bladder. Compared with lower urinary tract involvement, the pathophysiology of ketamine-associated upper urinary tract involvement is yet to be elucidated. Moreover, whether the occurrence of ketamine-associated hydronephrosis is caused by the same direct toxic effect or is secondary to lower urinary tract abnormality is still under debate. Since hydronephrosis appears to be involved later and less frequently, the secondary theory has been more accepted. A previous study showed that decreased functional bladder capacity was a risk factor of hydronephrosis development in chronic ketamine abusers, the result of which supports the secondary theory indirectly [22]. On this basis, some experts suggest that ketamine cystitis and ketamine hydronephrosis are characteristics of different clinical staging of ketamine-associated uropathy. However, there is a current challenge to this conclusion. Hopcroft et al. reported an evident change in ureteric interstitial metaplasia in a nephroureterectomy specimen from a chronic ketamine abuser [23]. Chang et al. described two chronic ketamine abusers whose ureteral wall tissue developed chronic inflammation alongside the formation of granulation tissue with inflammatory exudate [24]. Moreover, Jankovic et al. observed that the activation of NMDA inotropic receptors located on human ureteral smooth muscles could be responsible for muscle contraction [25]. These results provide possible evidence of the direct effect of ketamine on the upper urinary tract. We suggest that both direct and indirect mechanisms coexist and result in hydronephrosis.

Our study indicated that ketamine-associated hydronephrosis was a strong independent predictor for renal function decline or ESRD in these substance abusers, even after adjusting for other confounding factors including age, sex, and initial renal function. This result indicates that regardless of whether the initial renal function was normal or impaired, patients with hydronephrosis still have a worse renal prognosis. As previous studies mentioned, lower urinary tract symptoms usually appear first; the upper urinary tract is eventually involved, following the accumulation of ketamine and its metabolites in the urological system. Ketamine-associated hydronephrosis is usually bilateral; however, some unilateral cases have been reported [11]. The distension of renal calyces and pelvis causes anatomic and functional renal damage. Once hydronephrosis is detected, functional preservation intervention should be considered to relieve obstruction by percutaneous nephrostomy or insertion of double-J stents. According to the results, the interventions attained partial improvement of renal function initially; however, hydronephrosis would recur. Eventually, interventions demonstrated only limited benefits while the condition worsened, inevitably affecting renal function and leading to ESRD. Some reports suggest that ketamine withdrawal could prevent the progression of ketamine-associated uropathy; however, this was not observed in all patients [10,26,27]. A bladder capacity <150 mL might indicate a clinical threshold for conservative treatment failure and imply irreversible bladder damage [17]. Our study proposed that the occurrence of hydronephrosis is a potential indicator of irreversible renal damage in ketamine-associated uropathy. Thus, it is recommended that all chronic ketamine abusers with lower urinary tract symptoms are suggested to undergo ultrasonography to check for the presence of hydronephrosis. In addition, nephrologists are advised to regularly follow up with patients with ketamine-associated hydronephrosis, since they are at a higher risk of developing renal function decline than those without hydronephrosis.

This study revealed that elevated cholestatic liver enzyme levels correlated with the degree of ketamine-associated uropathy in these chronic ketamine abusers; this finding agrees with that of a previous study [22]. In 2009, Wong et al. first identified ketamine-associated hepatobiliary tract complications [28]. Since then, more similar cases have been reported. In a cohort study of 297 patients with ketamine-associated uropathy conducted in Hong Kong, the prevalence of liver injury was approximately 9.8% [29]. All cases were cholestatic, and the liver biopsy mainly showed bile duct injury, including common bile duct dilatation, microscopic bile duct injury, or even significant liver fibrosis. Another case series in Singapore showed marked elevation in alkaline phosphatase and gamma-glutamyl transferase levels in three chronic ketamine misuse patients [30]. The exact mechanism of ketamine-associated hepatobiliary injury remains unknown. Possible hypotheses included a ketamine-induced inflammatory process in the biliary tract or dysregulation of the biliary smooth muscle relaxation by ketamine. A study revealed that the dose and duration of ketamine use were not associated with the degree of liver function impairment [29]. However, Yee et al. reported that liver injury caused by ketamine occurs in a dose-dependent manner [22]. This study implies a possible correlation between the degree of cholestatic liver injury and the overall disease severity in chronic ketamine abusers. In addition, ketamine abuse should be considered in the differential diagnosis when assessing young patients with abnormal cholestatic liver injury.

This study has several limitations. First, poor compliance of the chronic ketamine abusers limited the follow-up. In total, 18 patients did not undergo ultrasonography, and 11 patients were not followed up with according to their renal function. Second, the study had a relatively small sample size; therefore, it may not have adequately detected other potential minor risk factors or further subgroup analysis. Third, these patients often tend to withhold true information regarding their substance abuse history; some difficulties were encountered while evaluating the duration and dosage of ketamine use. Nevertheless, to the best of our knowledge, this study was the first to evaluate the effects of ketamine-associated uropathy on renal health and longitudinal renal function follow-up in chronic ketamine abusers.

## 5. Conclusions

In conclusion, this study indicated that the occurrence of ketamine-associated hydronephrosis is a strong independent predictor for renal function decline in chronic ketamine abusers. Optimal imaging to detect abnormalities in these high-risk patients and referring potential candidates to a nephrologist for renal function follow-up are therefore mandatory. Moreover, ketamine abuse should be consistently included in the differential diagnosis when assessing young patients with unreasonable cholestatic liver injury, recurrent cystitis, or even hydronephrosis. Notably, more attention should be paid to ketamine-associated complications.

## Figures and Tables

**Table 1:** Demographic differences between patients with hydronephrosis and cystitis.

Parameter	All (*n* = 51)	Hydronephrosis (*n* = 22)	Cystitis (*n* = 29)	*p*-Value
Sex (male/female)	14/37	5/17	9/20	0.510
Age (years)	35.3 ± 5.1	34.6 ± 4.1	35.9 ± 5.8	0.365
Body height (cm)	162.2 ± 6.8	161.3 ± 7.6	163 ± 6.2	0.380
Body weight (kg)	52.9 ± 13.3	50.1 ± 13	55.1 ± 13.3	0.192
Body mass index (kg/m^2^)	19.9 ± 3.9	19.1 ± 3.6	20.6 ± 4.6	0.182
Systolic blood pressure (mmHg)	130.9 ± 25.9	135 ± 30.5	127.8 ± 21.9	0.329
Diastolic blood pressure (mmHg)	84.6 ± 17.9	88.8 ± 19.4	80.5 ± 17.9	0.101
Comorbidity				
Hypertension *	3 (6)	3 (14)	0	0.040
Type 2 DM	2 (4)	1 (5)	1 (3)	0.842
Renal function				
Initial BUN * (mg/dL)	18.8 ± 23	25.3 ± 29.4	10.7 ± 4.1	0.036
Initial creatinine * (mg/dL)	1.2 ± 1	1.6 ± 1.4	0.9 ± 0.4	0.041
Initial eGFR ** (mL/min)	83.5 ± 30	69.8 ± 34.5	93.9 ± 21.2	0.007
Final BUN ** (mg/dL)	32 ± 45.1	48.1 ± 55	11 ± 5.6	0.005
Final creatinine ** (mg/dL)	1.9 ± 2.6	3.2 ± 3.6	0.9 ± 0.3	0.007
Final eGFR ** (mL/min)	74.9 ± 38	48.5 ± 38.3	95 ± 22.5	<0.001
Renal outcome **	13 (25)	11 (50)	2 (7)	<0.001
ESRD	2 (4)	2 (9)	0	
eGFR decline >30%	11 (22)	9 (41)	2 (7)	
Follow-up duration (months)	36.7 ± 33	36.6 ± 32.2	36.7 ± 34.1	0.985
Liver function				
GOT (U/L)	133.6 ± 142.4	155.3 ± 137.2	117.2 ± 146.6	0.359
GPT (U/L)	172.8 ± 130	187.4 ± 98.7	162.2 ± 149.5	0.504
ALP ** (U/L)	499.1 ± 696.3	961.2 ± 870.6	152.5 ± 98.7	<0.001
GGT ** (U/L)	873.3 ± 802.3	1280 ± 890.3	443.9 ± 379	0.001
Total bilirubin (mg/dL)	1.2 ± 1.5	1.5 ± 1.6	1 ± 1.4	0.208

Values are expressed as mean ± standard deviation or number (%). * *p* < 0.05; ** *p* < 0.01. ALP, alkaline phosphatase; BUN, blood urea nitrogen; DM, diabetes mellitus; eGFR, estimated glomerular filtration rate; ESRD, end-stage renal disease; GGT, gamma-glutamyl transferase; GOT, glutamic oxaloacetic transaminase; GPT, glutamic pyruvic transaminase.

**Table 2:** Correlation analysis between renal function and liver function profile.

Parameter	Initial eGFR		Worse eGFR		Final eGFR	
	*^a^r*	*p*	*^a^r*	*p*	*^a^r*	*p*
GOT	−0.006	0.968	−0.275	0.055	−0.318	0.026
GPT	−0.039	0.785	−0.214	0.135	−0.220	0.125
ALP	−0.312	0.029	−0.698	<0.001	−0.580	<0.001
GGT	−0.414	0.011	−0.668	<0.001	−0.540	0.001

*^a^r* represents-Pearson correlation coefficient. ALP, alkaline phosphatase; eGFR, estimated glomerular filtration rate; GGT, gamma-glutamyl transferase; GOT, glutamic oxaloacetic transaminase; GPT, glutamic pyruvic transaminase.

**Table 3:** Multivariate analysis of risk factors associated with renal outcome among ketamine uropathy patients using a Cox proportional hazards model.

Parameter	Hazard Ratio	95% CI	*p*-Value
Hydronephrosis *	5.325	1.128–25.137	0.035
Age	0.923	0.790−1.077	0.309
Sex	1.469	0.290−7.431	0.642
BMI	0.821	0.635−1.061	0.132
Initial creatinine	0.950	0.581−1.553	0.838

The regressions coefficients and 95% coefficient interval value are indicated. All models are adjustments for the covariates above including hydronephrosis, age, sex, body mass index (BMI), and initial creatinine. * *p* < 0.05. CI, confidence interval.
